# Attempts to retreat from a dead-ended long capillary by backward swimming in *Paramecium*

**DOI:** 10.3389/fmicb.2014.00270

**Published:** 2014-06-11

**Authors:** Itsuki Kunita, Shigeru Kuroda, Kaito Ohki, Toshiyuki Nakagaki

**Affiliations:** ^1^Physical Ethology Laboratory, Research Institute for Electronic Science, Hokkaido UniversitySapporo, Hokkaido, Japan; ^2^Department of Complex and Intelligent Systems, Faculty of Systems Information Science, Future University HakodateHakodate, Japan; ^3^JST, CRESTTokyo, Japan

**Keywords:** *Paramecium*, long-term backward swimming, avoidance behavior, membrane potential, Ca^2+^ current, Hodgkin–Huxley-type model

## Abstract

We have observed how the ciliate *Paramecium* attempts to retreat from the dead-end of a long capillary that is too narrow for turning. After many trial-and-error episodes of short-term backward swimming (SBS), which is the conventional avoidance behavior exhibited in free swimming when an obstacle is faced, long-term backward swimming (LBS) that lasted five to ten times longer was developed. LBS may have a beneficial effect for complete withdrawal from the capillary space, although in our experiment it was impossible for the organism to do so due to the capillary length. In order to identify a physically possible mechanism for LBS, we propose model equations for the membrane potential of Hodgkin–Huxley type, which describe the control of ciliary movement. The physiological implications and physical mechanism of the development of LBS are discussed.

## 1. Introduction

It is not trivial to study how unicellular organisms tackle a problem, as they often try different behaviors instead of previously unsuccessful behaviors to solve dilemmas. One of the most important issues to be addressed here is what types of behavior can be induced and how such behavioral options appear in terms of information processing in the cell. It is interesting to identify whether mechanisms of information processing are based on mechanical equations of motion, because in some sense the physical basis of an adaptation or learning process could be suggested (Corning et al., 1973; Bray, 2009).

Aneural organisms such as protozoa and plants have been well studied within the context of neurobiology (Eisenstein, 1975). Comparative studies show the similarity among them; learning and habitation behaviors often develop in ciliates and plants in response to external stimulation and environmental conditions. *Paramecium* and *Stentor* are some of the most well-studied model microorganisms. The excitable cell membrane of Paramecium bears interesting similarity to that of neurons in higher animals (Hamilton, 1975; Wood, 1975).

Based on many electrophysiological studies, dynamic changes in membrane potential in *Paramecium* can be described by the highly non-linear differential equations of the Hodgkin–Huxley type, which was originally proposed for neuron in squid. For *Paramecium*, some of the equations of motion relevant to the membrane potential have already been well established; they involve the electrophysiological properties of the potassium and calcium channels, which are the main contributors to the membrane potential in *Paramecium*.

In fact, the membrane potential is closely related to swimming speed and direction in *Paramecium*. The swimming obeys collective motion of beating cilia and this collective motion is regulated by the membrane potential. Y. Naitoh concludes that the ciliary motion and the membrane potential are nearly in one-to-one-correspondence (Naitoh and Sugino, 1984). An important question arises then: is the mechanism of learning and habitation understood by means of Hodgkin–Huxley type equations for membrane potential in *Paramecium*? Here we will consider the question, shedding light on a retreating behavior from a capillary space. An answer obtained in this report is positive.

When a forward swimming *Paramecium* collides with a solid object, the specimen first swims backward for a short distance because the beating direction of the cilia is temporarily reversed (Eckert, 1972; Naitoh, 1974; Naitoh and Sugino, 1984). The cilia then gradually resume beating in their original direction, and the specimen begins to swim forward again. This behavioral response is known as the avoidance response, which is an innate reflex action. In the early 1900s, Smith (1908) and Day and Bentley (1911) observed the swimming of *Paramecium* in a capillary tube. The *Paramecium* initially showed a simple avoidance response at the closed end of capillary tube, because it was difficult for the organism to turn around in the narrow space of the capillary. After this avoidance action had been repeated a number of times, the *Paramecium* exhibited novel behavior; the organism folded up its own body very tightly and successfully turned in the confined space. This observation subsequently attracted much attention from researchers because it implied the development of new behavior. Development of new behaviors had been only observed for higher animals and was believed to be a characteristic of intelligence (St John and Corning, 1973).

It is known that backward swimming in *Paramecium* depends only on depolarization of the electrical potential in the cellular membrane (Eckert, 1972). More specifically, the Ca^2+^ current can reverse the rotation of the ciliary beat and thus regulate the duration of backward swimming. The relationship between this behavior and the membrane potential in *Paramecium* has been studied using electrophysiological measurements by Naitoh (1974); Naitoh and Sugino (1984).

In this report, we describe how the ciliate *Paramecium* attempted to retreat from the dead-end of a capillary that was too narrow in which to turn. In addition to the conventional avoidance behavior of short-term backward swimming (SBS) when an obstacle is faced during free swimming, we find emergent new avoidance behavior: long-term backward swimming (LBS) that lasted five to ten times longer than SBS. We next analyze the dynamical properties of the model equation for the membrane potential and consider a possible mechanism for the LBS. Finally, we discuss the physiological implications of episodes of SBS and LBS and propose a physical mechanism for the development of these two types of behavior.

## 2. Organisms and experimental methods

Specimens of *Paramecium* were grown at room temperature in a decocted liquid extracted from straw. This was exchanged with the assay medium [1.0 mM Tris-HCl (pH = 7.2), 1.0 mM CaCl_2_, 2.0–20.0 mM KCl] by using a narrow glass pipette 3 h before the test.

**Figure 1:**
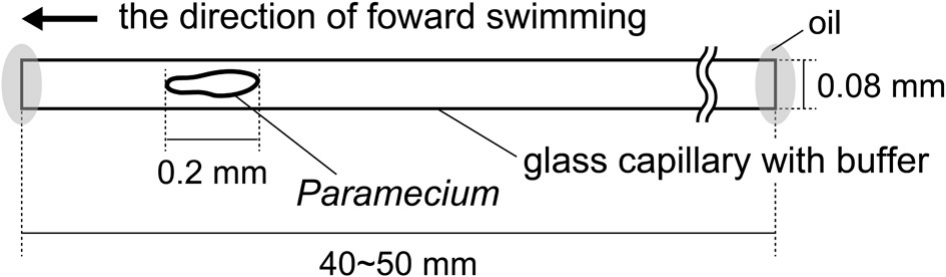
**Experimental setup**.

Figure 1 shows the experimental setup used for the behavioral test of *Paramecium* in a dead-ended capillary of length 40–50 mm. The capillary had an internal diameter of 0.08 mm, which is about twice the width of the protozoan body, and was used in the horizontal orientation. At the beginning of a trial, an individual *Paramecium* was collected with a narrow glass pipet and placed in the capillary filled with the assay medium. The ends of the capillary were then closed with mineral oil in order to confine the *Paramecium* inside. Each specimen was used only once in all experiments.

The specimens were observed using a stereomicroscope (Olympus SZX16). Microscopic video images were taken using a CCD camera and recorded by a video camera. The video images were saved in AVI format on a personal computer before analysis with the free software NIH imageJ.

## 3. Behavior of *Paramecium* in a dead-ended capillary

### 3.1. Two types of backward swimming of *Paramecium* in a dead-ended capillary

**Figure 2:**
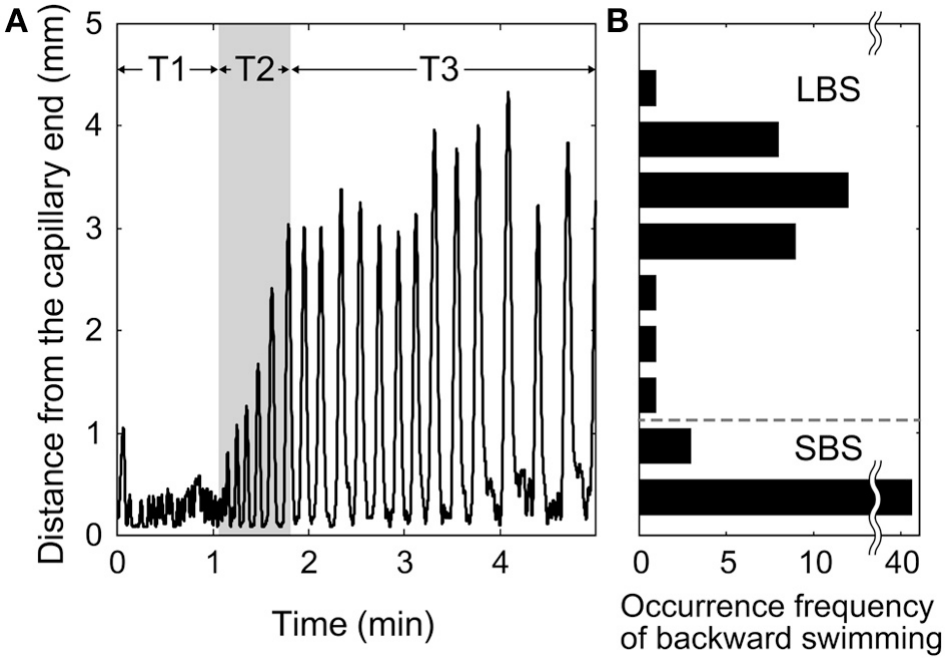
**Typical behavior of *Paramecium* in a dead-ended capillary**. **(A)** Time-course of distance from capillary end. **(B)** Distribution of backward swimming distances.

Figure 2 shows typical behavior of a specimen in a dead-ended capillary [(KCl) = 4 mM]. Figure 2A shows a time-course of the distance from the capillary end. When the forward swimming *Paramecium* bumped against the capillary end, the specimen began to swim backward before reversing direction and swimming forward again. This behavior was repeated many times. Shortly after the first collision with the capillary end (in the region labeled T1 in Figure 2), the backward swimming distance was 0.3–0.5 mm, which corresponds to one to two times the length of the body. The period of backward swimming was 2–5 s. The backward swimming distance gradually became longer (region T2 in Figure 2A), and the period increased to 5–10 s. Finally, in region T3 in Figure 2A, the distance reached 3–4 mm and the period increased to 5–15 s. Figure 2B shows the distribution of backward swimming distances in Figure 2A. Two peaks are present at 0.3–0.5 mm and 3–4 mm. This behavior was observed in 13 of 15 tested individuals under the same experimental conditions.

These results imply that *Paramecium* exhibits two types of backward swimming, which are distinguishable by their distances and periods. The first type can be referred to as short-term backward swimming (SBS) with distances of 0.3–1 mm and periods of 2–10 s. The second type is long-term backward swimming (LBS) with distances of 3–4 mm and periods of 10–15 s.

### 3.2. Effect of long-term backward swimming on concentration of potassium ions

**Figure 3:**
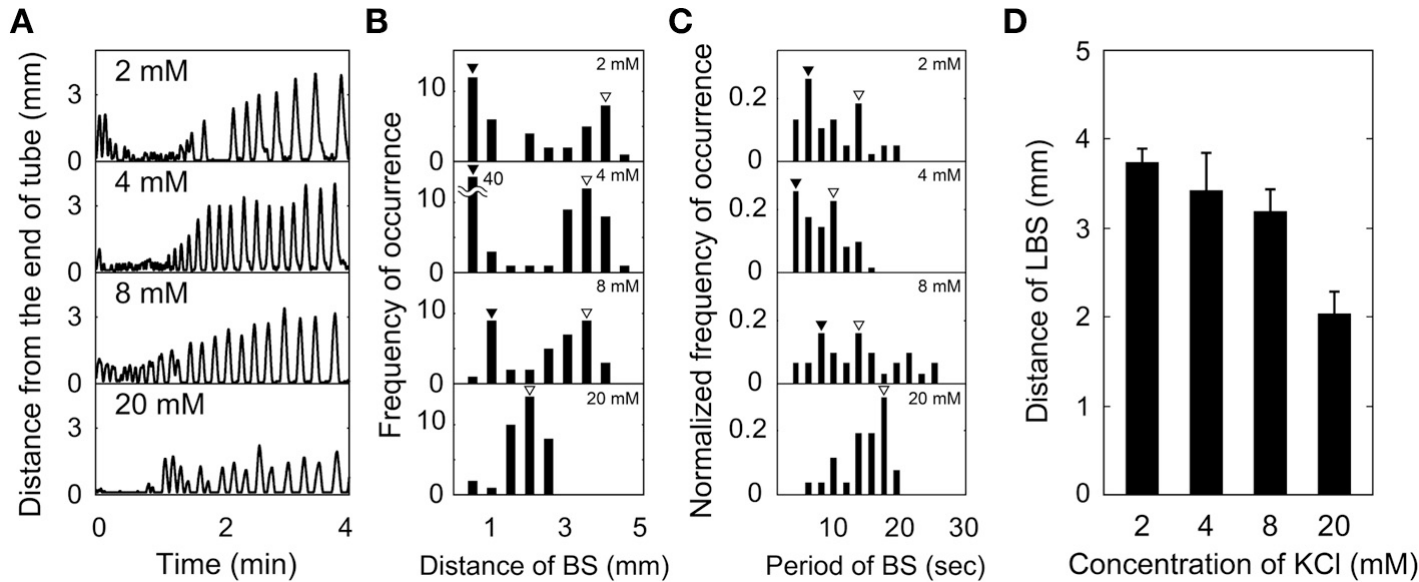
**Effect of potassium ion concentration on backward swimming (BS) of *Paramecium* in a dead-ended capillary**. **(A)** Time-courses of distance from capillary end. **(B)** Distribution of backward swimming distances. **(C)** Distribution of time periods of backward swimming. **(D)** Dependence of LBS distance on potassium ion concentration. **(B–D)** The number of individuals at 2, 4, 8, and 20 mM of potassium ions was 3, 13, 3, and 3, respectively. Open and closed triangles show the peak of SBS and LBS, respectively.

**Figure 4:**
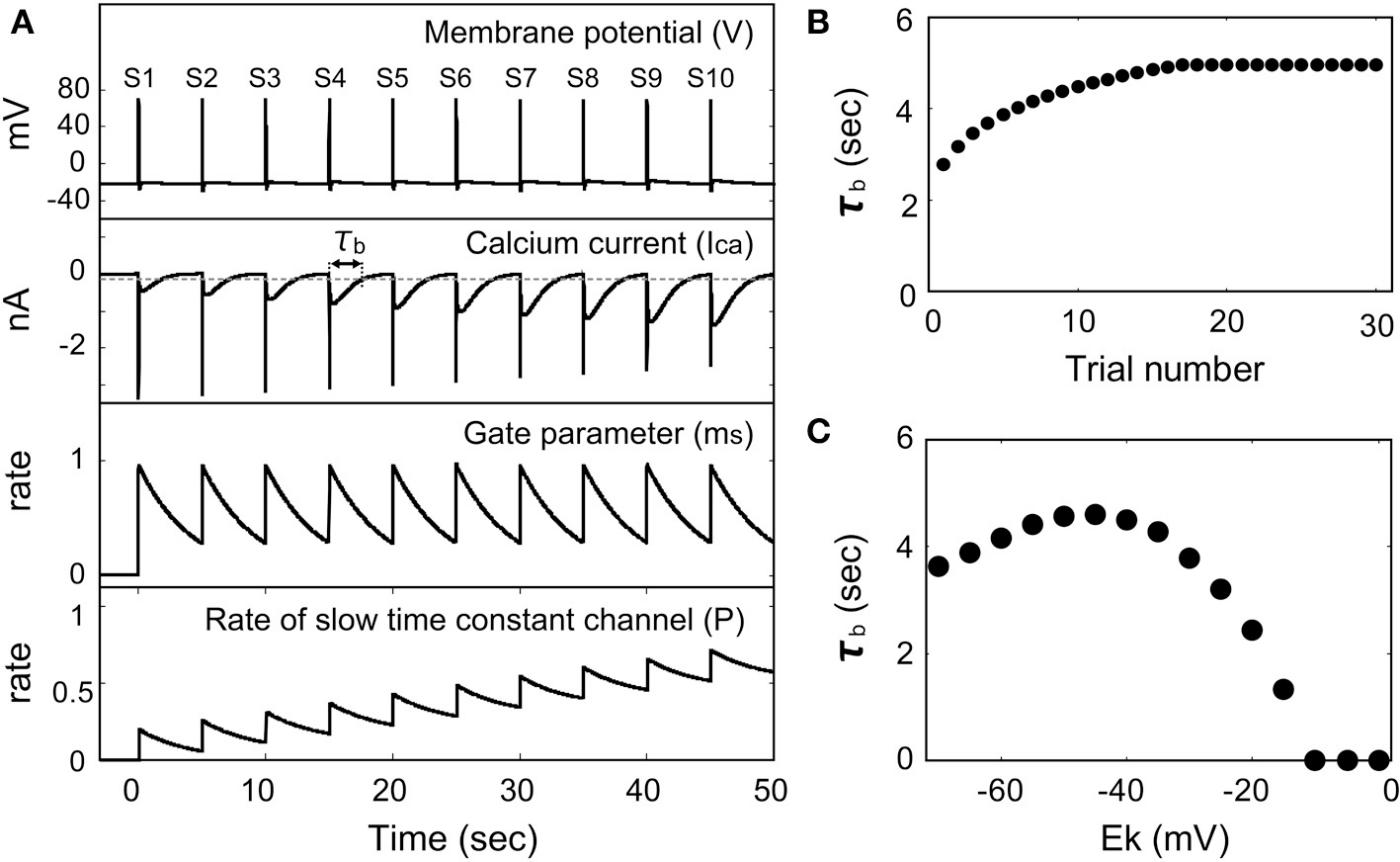
**Membrane potential response induced by a periodic outward flow of current in the presence of a Ca^2+^ channel with the slow time constant. (A)** Time dependence of the membrane potential, Ca^2+^ current, gate parameter (*m*_*s*_), and the rate of the channel with the slow time constant (*E*_*K*_ = −40 mV). **(B)** Dependence of the Ca^2+^ current duration time on the number of trials (*E*_*K*_ = −40 mV). **(C)** Dependence of the Ca^2+^ current duration time on the potassium equilibrium potential.

Figure 3 shows effect of potassium ion concentration (2, 4, 8, and 20 mM) on backward swimming of *Paramecium* in a dead-ended capillary. Figure 3A shows the trajectory of backward and forward swimming motions in the time and one-dimensional space plot, and the distance of backward swimming was measured from the swimming trajectory. The statistical occurrence of this distance was shown in Figure 3B. They had peaks at 0.3–1 mm (open triangles in Figure 3B) and 3–4 mm (closed triangles in Figure 3B) at 2, 4, and 8 mM, and there was a single peak at 2 mm (closed triangles in Figure 3B) at 20 mM. In Figure 3C, the distribution of backward swimming periods is shown for each K^+^ concentration; at 2, 4, and 8 mM there were peaks at 3–9 s (open triangles in Figure 3C), and 10–15 s (closed triangles in Figure 3C), and at 20 mM there was a single peak at 17 s (closed triangles in Figure 3C). Figure 3D shows the dependency of LBS distance on potassium ion concentration. The LBS distance decreased with increasing concentration.

In summary, *Paramecium* in a dead-ended capillary exhibited both SBS and LBS at K^+^ concentrations of 2–8 mM, and only LBS at 20 mM.

## 4. Mathematical modeling of long-term backward swimming in *Paramecium*

### 4.1. Behavioral model for *Paramecium* based on Hodgkin–Huxley-type equation

The experimental results described above provide clear evidence that repeated mechanical stimulus in a dead-ended capillary can increase the distance of backward swimming in *Paramecium*. The modification of behavior in *Paramecium* is a novel behavior, which develops from the restriction of the moving region. This implies that there is an adaptive capacity for spatial navigation in *Paramecium*. The important thing for understanding such behaviors or capacities in organisms is an understanding of the physiological mechanism.

The movement of cilia occurs due to the ciliary motion. The ciliary motion is regulated by the membrane potential change or the biochemical reactions in the cell and the cilium. In *Paramecium* the relationship between the behaviors and the membrane potential changes has been well studied (Eckert, 1972; Naitoh, 1974; Naitoh and Sugino, 1984). Therefore, we now attempt to understand the underlying mechanisms of these behaviors in *Paramecium* through mathematical modeling of membrane potential change.

Based on the Hodgkin–Huxley-type model for the excitation dynamics of membrane potentials (Hodgkin and Huxley, 1952; Naitoh and Sugino, 1984), we propose a simplified model to explain the behavior observed in our biological experiment. In the mathematical modeling that follows we consider only the case of LBS, even though the experiments also revealed SBS. Before constructing the model, we explain the mechanism of the membrane potential response in *Paramecium* induced by mechanical stimulus.

The swimming behavior of *Paramecium* depends on its ciliary motion, which is in turn controlled by a membrane potential caused mainly by the difference in concentration of Ca^2+^ and K^+^ between the interior and exterior of the cell. The mechanism by which the membrane potential changes is essentially the same as that in nerve cells and muscle cells, although the ionic species involved are different.

When the forward swimming *Paramecium* collides with a solid object, extracellular Ca^2+^ ions flow into the cell, mediated by opening of the mechano-sensitive Ca^2+^ channels that are distributed in the anterior region of the cell (Ogura and Machemer, 1980; Satow et al., 1983; Machemer and Machemer-Röhnisch, 1984; Tominaga and Naitoh, 1994). This depolarizes the membrane potential. The depolarized membrane is more permeable to Ca^2+^ ions due to opening of the voltage-sensitive Ca^2+^ channels, which are localized in cilia. This results in a large regenerative depolarization (Dunlap, 1977; Machemer and Ogura, 1979). The increased concentration of Ca^2+^ in cilia leads to the reversal of ciliary beating and hence to backward swimming. The increased membrane potential also opens the voltage-sensitive K^+^ channels, allowing intracellular K^+^ ions to flow out of the cell. This outflow leads to repolarization of the membrane. The cilia gradually resume their original direction of beating as the membrane potential decreases, and the *Paramecium* begins to swim forward again. The duration time of backward swimming corresponds to the duration time of Ca^2+^ current flow.

Naitoh and Sugino (1984) have reported that the avoidance response of *Paramecium*, which corresponds to SBS in our report, can be represented by a Hodgkin–Huxley-type equation on the basis of the above scenario for the physiological mechanism of backward swimming. However, the mechanism of the membrane response to repeated current stimuli is unclear. Naitoh (1990) has suggested that Ca^2+^ channels with slow time constants (which deactivate slowly) might carry Ca^2+^ current after the action potential has gone, even though Ca^2+^ channels with fast and slow time constants are the same physically. Hinrichsen et al. (1984) reported that the backward swimming of a mutant specimen of *Paramecium*, whose Ca^2+^ current deactivated poorly, persisted for longer under high concentrations of K^+^ ions than that of wild-type *Paramecium*.

According to this line of understanding, we can assume that the time constants of the Ca^2+^ channels in *Paramecium* can be changed by the stimulus. LBS is induced by the Ca^2+^ current, which is deactivated slowly due to Ca^2+^ channels with slow time constants that remain activated after the action potential is gone. We also assume that the membrane potential in the cell corresponds to that in the cilia without regard to the localization of Ca^2+^ channels for simplicity. We formulated an equation for the membrane potential relevant to LBS in *Paramecium* (regions T2 and T3 in Figure 2A). The time-course of the membrane potential can be expressed as:

(1) $$\begin{array}{l} C_{m}\dot{V}\left(t\right)=\delta \left(t\right)I_{app}\left(t\right)-I_{Ca}\left(t,\;V\right)\\ \;-I_{K}\left(t,\;V\right)-I_{leak}\left(t,\;V\right). \end{array}$$

Here *t* is the time, *C*_*m*_ is the membrane capacitance, δ is a function that switches the outward current, and *I*_*app*_(*t*) is the outward electric current, which corresponds to the mechanical stimulus applied by bumping against the end of the capillary in our experiment. *I*_*Ca*_ is the Ca^2+^ current, *I*_*K*_ is the K^+^ current, and *I*_*leak*_ is the leak current. These three quantities can be expressed as:

(2) $$I_{Ca}\left(t,V\right)=g_{Ca}\left(V\left(t\right)-E_{Ca}\right),$$

(3) $$I_{K}\left(t,V\right)=g_{K}\left(V\left(t\right)-E_{K}\right),$$

(4) $$I_{leak}\left(t,V\right)={\bar{g}}_{leak}\left(V\left(t\right)-E_{leak}\right),$$

where *g*_*Ca*_ and *g*_*K*_ are the ionic conductance (the rate of passage of ions), the definition of which will be given later. *g*_*leak*_ is the maximum ionic conductance of the leak ion channel. *E*_*Ca*_ and *E*_*K*_ are the equilibrium potentials of Ca^2+^ and K^+^, respectively. These two quantities can be described by the Nernst equation (Equation 5) for the diffusion of electric charge. *E*_*leak*_ is the equilibrium potential of the leak ions, and is given approximately by Equation (6).

(5) $$E_{X}=\left(RT/zF\right)\ln\text{​}\left([X^{z+}]o/[X^{z+}]i\right)\;\left(X\in \{Ca,K\}\right),$$

(6) $$E_{leak}=\left({\bar{g}}_{LCa}E_{Ca}+{\bar{g}}_{LK}E_{K}\right)/\left({\bar{g}}_{LCa}+{\bar{g}}_{LK}\right).$$

Here, *R* is a gas constant, *T* is the absolute temperature, *z* is the ionic valence, and *F* is the Faraday constant. The parameters [*X*^*z*+^]_*o*_ and [*X*^*z*+^]_*i*_ are the extracellular and intracellular concentrations of *X*^*z*+^ ions, respectively. *g*_*LCa*_ and *g*_*LK*_ are the ionic conductance of the Ca^2+^ and K^+^ channels, which are insensitive to changes in the membrane potential.

The Ca^2+^ and K^+^ currents are controlled by the conductance of the Ca^2+^ and K^+^ channels, respectively. The Ca^2+^ conductance (*g*_*Ca*_) is described using the activation gate factor *m* and the deactivation gate factor *h*. The K^+^ conductance (*g*_*K*_) is described by the activation gate factor *n*. These factors are the variables that represent the gating of the voltage-sensitive ionic channels.

The occurrence of LBS requires long-term activation of the Ca^2+^ channel, because the duration time of backward swimming in *Paramecium* depends on the time for which Ca^2+^ current flows. We assume that the Ca^2+^ channels are in one of two modes, with a fast or slow time constant with respect to the deactivation process. We now introduce two more parameters, *g*_*CaS*_ and *P*(*t*), to represent the conductance and occurrence rate of Ca^2+^ channels with the slow time constant, respectively. We include these parameters in the original equation for Ca^2+^ conductance in the Hodgkin–Huxley-type model for *Paramecium* reported by Naitoh and Sugino (1984). The definition of *P*(*t*) will be given later. The total Ca^2+^ conductance is given as the sum of the products of the occurrence rate with the conductance of the Ca^2+^ channels with fast and slow time constants. The resulting equations for ionic conductance are as follows:

(7) $$g_{Ca}\left(t,\;V\right)=\left(1-P\left(t\right)\right)g_{CaF}\left(t,\;V\right)+P\left(t\right)g_{CaS}\left(t,\;V\right),$$

(8) $$g_{CaF}\left(t,\;V\right)={\bar{g}}_{Ca}m_{F}{\left(V\right)}^{5}\{1-{\left(1-h_{F}\left(V\right)\right)}^{5}\},$$

(9) $$g_{CaS}\left(t,\;V\right)={\bar{g}}_{Ca}m_{S}{\left(V\right)}^{5}\{1-{\left(1-h_{S}\left(V\right)\right)}^{5}\},$$

(10) $$g_{K}\left(t,V\right)={\bar{g}}_{K}n\left(V\right),$$

where *g*_*CaF*_ is the Ca^2+^ conductance with the fast time constant. *g*_*Ca*_ and *g*_*K*_ are the maximum Ca^2+^ and K^+^ conductance of the membrane when all the Ca^2+^ and K^+^ channels are activated, respectively. *j*_*F*_ and *j*_*S*_ (*j* ∈ {*m*, *h*}) are the gate variables for the channels with fast and slow time constants. The equations for the gate variables are as follows:

(11) $$\begin{array}{l} \dot{x}=\alpha_{x}\left(V\right)\left(1-x\right)-\beta_{x}\left(V\right)x,\\ \;x\in \{m_{F},\;m_{S},\;h_{F},\;h_{S},\;n\}, \end{array}$$

where *x* is the opening rate of the gates. α_*x*_ and β_*x*_ are functions of the membrane potential, which are determined by fitting the rate constants using analytical expressions as follows:

(12) $$\begin{array}{l} \alpha_{m_{F}}\left(V\right)=-0.0224\left(V+2.5809\right)\\ \;/\left(\exp\text{​}\left(-\left(V+2.5809\right)/0.7331\right)-1\right), \end{array}$$

(13) $$\beta_{m_{F}}\left(V\right)=0.1426\exp\text{​}\left(-V/39.3464\right),$$

(14) $$\alpha_{h_{F}}\left(V\right)=0.1\exp\text{​}\left(-\left(V+30\right)/5\right),$$

(15) $$\beta_{h_{F}}\left(V\right)=1/\left(\exp\text{​}\left(\left(38.2866-V\right)/30.9397\right)+1\right),$$

(16) $$\begin{array}{l} \alpha_{n}\left(V\right)=0.0375\left(58.5845-V\right)\\ \;/\left(\exp\text{​}\left(\left(58.5845-V\right)/8.16699\right)-1\right), \end{array}$$

(17) $$\beta_{n}\left(V\right)=0.1015\exp\text{​}\left(-V/68.0968\right),$$

(18) $$\begin{array}{l} \alpha_{j_{S}}\left(V\right)=\alpha_{j_{F}}\left(V\right)\gamma ,\;\beta_{j_{S}}\left(V\right)\;=\;\beta_{j_{F}}\left(V\right)\gamma ,\\ \;j\in \{m,h\}, \end{array}$$

(19) $$\gamma =\{\begin{array}{ll} \bar{\gamma } & \delta =0,\\ 1 & \delta =1. \end{array}$$

Here, γ (0 ≤ *γ* ≤ 1) is a constant. For each fixed *V* in Equation (11), the time constants (τ_*x*_(*x* ∈ {*m_F_*, *m_S_*, *h_F_*, *h_S_*, *n*})) and equilibrium values (*x*_∞_(*x* ∈ {*m_F_*, *m_S_*, *h_F_*, *h_S_*, *n*})) of the gate variables are given as follows:

(20) $$\tau_{x}=1/\left(\alpha_{x}+\beta_{x}\right),\;x_{\infty }=\alpha_{x}/\left(\alpha_{x}+\beta_{x}\right).$$

*P* (0 ≤ *P* ≤ 1) is the rate of the Ca^2+^ channel with the slow time constant in the deactivation process. The Ca^2+^ channels with the fast and slow time constants are the same physically, and it is assumed that the mode of the Ca^2+^ channel is changed by the stimulus-induced membrane potential response. The relevant equation is as follows:

(21) $$P\left(t\right)=\left(P\left(t_{i}\right)+\Delta P\right)\exp\text{​}\left(-\left(t-t_{i}\right)/\tau_{P}\right),$$

where *t*_*i*_ is the finishing time of the *i*^*th*^ stimulation, Δ*P* is the maximum rate of the Ca^2+^ channel changing the mode, and τ_*P*_ is the decay time constant of *P*.

The numerical parameters and initial values for our proposed model are based on the experimental conditions and the measurements of membrane potential reported by Naitoh *et al* and are as follows: *C*_*m*_ = 2 [μF/cm^2^], *E*_*Ca*_ = 125 [mV] ([Ca^2+^]_*i*_ = 6.06 × 10^−8^ [M], [Ca^2+^]_*o*_ = 10^−3^ [M]), *E*_*K*_ = −57 ~ 0 [mV] ([K^+^]_*i*_ = 20 [mM], [K^+^]_*o*_ = 2–20 [mM]), *g*_*LCa*_ = 0.1 [mS/cm^2^], *g*_*LK*_ = 0.8 [mS/cm^2^], *T* = 298 [K], *R* = 2.0 (cal/K·mole), *F* = 23,000 (cal/V·mole), *g*_*Ca*_ = 0.6667 [mS/cm^2^], *g*_*K*_ = 1.3333 [mS/cm^2^], *g*_*leak*_ = 0.42 [mS/cm^2^], *γ* = 0.005–0.05, *C*_*P*_ = 0.2, τ_*P*_ = 700.0, *V*(0) = −30 [mV], *m*_*F*_(*V*(0)) = 0.2911, *m*_*S*_(*V*(0)) = 0.01, *h*_*F*_(*V*(0)) = 0.162, *h*_*S*_(*V*(0)) = 0.1, *n*(*V*(0)) = 0.0163, *P*(0) = 0, *I*_*app*_ = 100.

### 4.2. Simulation of proposed model for backward swimming in *Paramecium*

We simulated the membrane potential responses when the outward electric current is injected periodically, because LBS occurred by repeating the colliding with the end of capillary. Figure 4A shows time-courses of the membrane potential, the Ca^2+^ current, the gate parameter *m*_*S*_, and the rate of the Ca^2+^ channel with the slow time constant when the outward electric current is injected periodically. Here it was assumed that [K^+^] = 4 mM (*E*_*K*_ = −40 mV). When the outward electric current was injected before all the Ca^2+^ channels with the slow time constant had returned to the mode with the fast time constant (*P* > 0), the rate of the Ca^2+^ channel with the slow time constant increased gradually. This resulted in an increase in magnitude of the Ca^2+^ current after the action potential had passed.

Figure 4B shows the dependency of the duration for which Ca^2+^ current flows (τ_*b*_) on the number of trials (the number of collisions with the capillary end). The duration time is defined as the time for which the Ca^2+^ current is smaller than a threshold value. The duration time increased with the number of trials and eventually became saturated. The time for which Ca^2+^ current flows corresponds to the duration time of backward swimming in *Paramecium*, and the results of this simulation represent the behavior displayed during regions T2 and T3 in Figure 2A.

Figure 4C shows the dependency of the duration time of Ca^2+^ current flow on the equilibrium potential of the K^+^ ion. The threshold for this duration time was set to −0.1 (nA) based on the experimental data reported by Naitoh and Kaneko (1972). The duration time is maximum at *E*_*K*_ = −40 mV, which corresponds to [K^+^] = 4 mM.

The maximum value of the duration time of Ca^2+^ current flow depends on the time constants of the gate parameters and on the threshold value of the duration time (data not shown). As shown in Figure 6B, the time constant of *h*, which influences the duration time of Ca^2+^ current flow, reached its maximum value when the membrane potential was −20 mV, which is the same value of the resting potential as *E*_*K*_ and *E*_*Ca*_ were −40 and −125 mV, respectively. Therefore, when the threshold value for the duration time is sufficiently low, the Ca^2+^ current-flow duration time is maximum at *E*_*K*_ = −40 mV as shown in Figure 4C. When the threshold value for the duration time is sufficiently high, the Ca^2+^ current-flow duration time decreases with increasing *E*_*K*_. The latter case was experimentally observed, as illustrated by the result in Figure 3D.

### 4.3. Mathematical mechanism for development of LBS

To understand the mechanism of the long-term Ca^2+^ current, which is invoked by the Ca^2+^ channel with slow time constant, we analyzed the relationship between the change of membrane potential and the move of nullcline.

**Figure 5:**
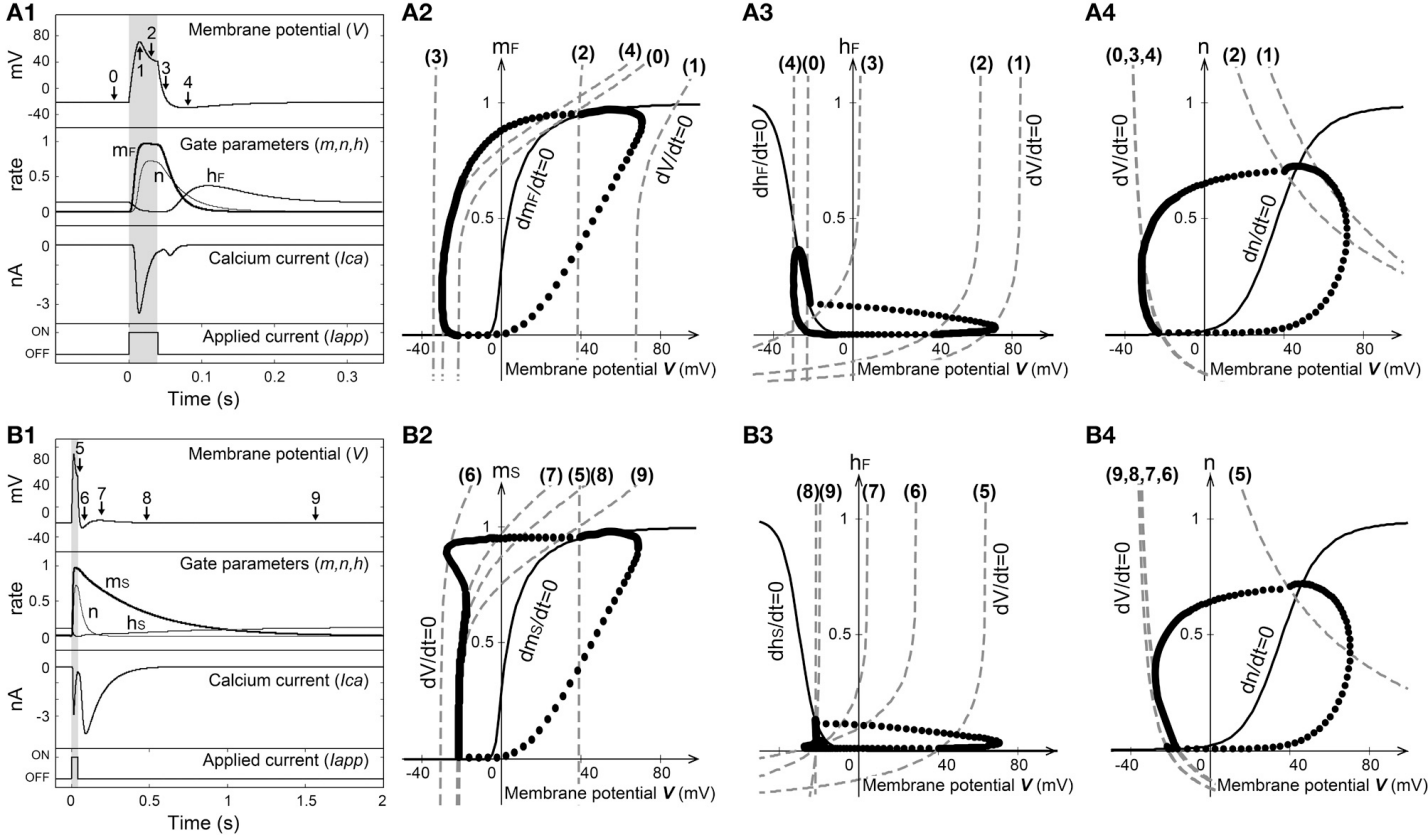
**Stimulus-induced membrane potential response in the absence (A) and presence (B) of a Ca^2+^ channel with the slow time constant (*E_K_* = −40 mV)**. (1) Time-courses of the membrane potential, gate parameters, and Ca^2+^ current. (2–4) Phase-plane plots of *m*, *h*, and *n* versus the membrane potential. The dashed lines represent the *V* nullcline, the solid lines represent the gate parameter (*m, h, n*) nullcline, and the solid circles indicate the orbits of solution obtained by numerical simulation.

**Figure 6:**
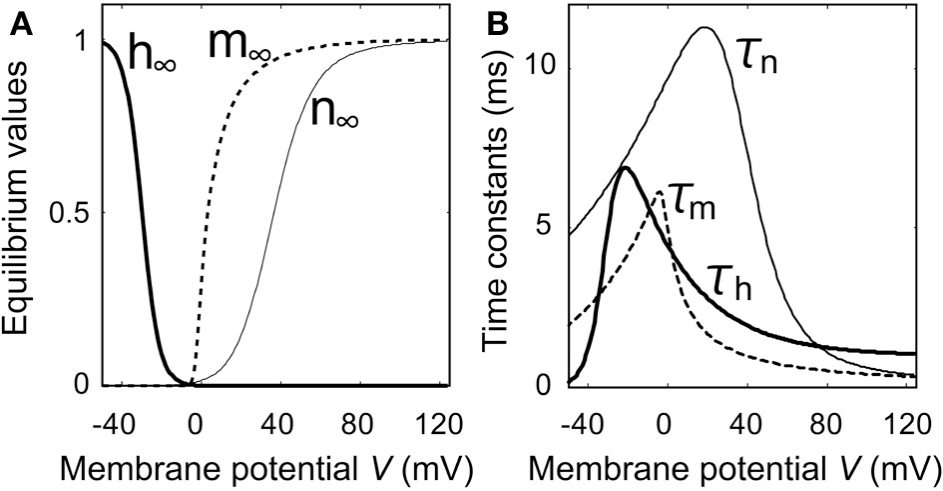
**Characteristics of gate parameters as a function of membrane potential**. **(A)** Equilibrium values of gate parameters. **(B)** Time constants of gate parameters.

Figures 5A,B show the stimulus-induced membrane potential responses in the absence (P = 0) and presence (P = 1) of Ca^2+^ channels that have slow time constants with respect to the deactivation process. Figure 6 shows the dependence of the gate parameters on the membrane potential.

The injection of an outward electric current into the cell produced a passive exponential membrane depolarization, and a *V* nullcline ($\dot{V}$ = 0) that was shifted from (0) to (1) in Figure 5A. As the time constant of *m*_*F*_ was smaller than those of *h*_*F*_ and *n* (Figure 6B), both *m*_*F*_ and *n* increased as the membrane potential became positive, whereas *h*_*F*_ decreased (Figure 6A). *m*_*F*_ increased before changes occurred in *h*_*F*_ and *n*. Here Ca^2+^ ions flow into the cell when the Ca^2+^ channels are opened. The magnitude of the membrane potential increased with the magnitude of the Ca^2+^ current.

When the membrane potential became larger than 20 mV, *n* began to increase as shown in Figure 6A. Now K^+^ ions flow out of the cell due to the opening of the K^+^ channels. The magnitude of the membrane potential was controlled by increasing the magnitude of the K^+^ current. When the membrane potential approached *E*_*Ca*_, it began to decrease because the magnitude of the Ca^2+^ current was small and the magnitude of the K^+^ current was large. Although the *V* nullcline then shifted from (1) to (2), there was little change in the equilibrium values of the gate parameters (*m*_*F*_, *h*_*F*_, *n*) when the magnitude of the outward electric current was large enough. Therefore, the Ca^2+^ channels remained in the activated state.

The membrane potential decreased sharply after the outward electric current was gone, and then the *V* nullcline shifted from (2) to (3). As the time constant of *m*_*F*_ was smaller than those of *h*_*F*_ and *n*, the decrease of *m*_*F*_ occurred before the decrease of *n* and the increase of *h*_*F*_. The K^+^ current was still carried after the Ca^2+^ current had stopped, and the membrane potential became hyperpolarized. The *V* nullcline then shifted from (3) to (4), and the membrane potential neared the resting state. *Paramecium* swims backward while Ca^2+^ current flows due to activation of the Ca^2+^ channels.

As shown in Figure 5B, the *V* nullclines shifted successively from (5) to (9) in the presence of the Ca^2+^ channel with the slow time constant (*P* = 1). In Figure 5B2 it is apparent that the orbit of the solution was deformed due to the slow time constants of *m*_*S*_ and *h*_*S*_ in the deactivation process of the Ca^2+^ channel. As the time constants of *m*_*S*_ and *h*_*S*_ were larger than that of *n*, the Ca^2+^ current still flowed due to the slow convergence of *m* and *h* after the K^+^ current had stopped. Although the Ca^2+^ current flowed while the Ca^2+^ channels were activated, because the Ca^2+^ current was small [(7–8) in Figure 5B], the change in membrane potential was also small. Therefore, given the presence of Ca^2+^ channels with the slow time constant, our proposed model reproduces the Ca^2+^ current that invokes backward swimming in *Paramecium* after the action potential is gone.

## 5. Discussion

The relationship between the membrane potential, which invokes ciliary motion, and the behavior of *Paramecium* is well studied. Therefore, the behavior of *Paramecium* is a good example for the study of physical mechanisms of behavior in organisms in general. In this report, we have analyzed the mechanism of LBS using mechanical equations.

Our experiment shows that two types backward swimming are exhibited (SBS and LBS) when *Paramecium* bumps against the end of the capillary. As SBS is the short-term response with a period in the range 1–2 s, this is the conventional avoidance response when an obstacle is faced during free swimming. LBS is the long-term response with a period of 5–15 s, and thus represents a novel type of avoidance behavior. We have shown that LBS is invoked by the long-term activation of Ca^2+^ channels, because LBS occurred even when the potassium equilibrium potential was zero. Our proposed mathematical model reproduces the experimentally observed behavior.

Our mathematical modeling allows us to discuss possible mechanisms for the development of LBS in *Paramecium*. The Hodgkin–Huxley-type equation that describes the membrane potential for behavior in *Paramecium* does not obey the all-or-nothing law such as that in nerve cells because the equation has one stable state, which depends on the extracellular ionic concentration and the presence or absence of an outward stimulus current. Although one might consider that the membrane potential response can easily be determined, in practice a number of different responses can be expected due to the presence of three variables with different time scales in the non-steady state. We have shown that the long-term non-steady state imposed by the presence of Ca^2+^ channels with a slow time constant in the deactivation process invoke LBS, which is caused by an increase in the duration time of Ca^2+^ current flow.

The properties of time constants related to Ca^2+^ channels in living organisms are unclear. However, channel proteins have rather complex structures and are involved in a number of different reactions, thus it is feasible that Ca^2+^ channels can have slow time constants. Although the experimental conditions differ from our experiment, Hennessey and Kung (1985) have reported that the inactivation of Ca^2+^ currents in *Paramecium* shows fast (10 ms range) and slow (10 s range) kinetics. It is expected that these properties will become more clearer through further electrophysiological measurements and the study of molecular dynamics.

Our model proposes that the development of LBS is invoked by a change in the Ca^2+^ channel from the fast time-constant mode in the deactivation process to the slow time-constant mode. As this is a reversible physiological change, LBS in *Paramecium* is a type of adaptive behavior in a narrow space. We suggest that small changes in parameters such as the time constant of channel proteins enable the organism to adapt when confronted with problematic conditions.

We now briefly discuss the biological implications of LBS, which represents novel behavior for retreat from a dead-ended capillary and is induced by repeated collisions with the end of the capillary. Although SBS facilitates the avoidance of obstacles, obstacles cannot be avoided in all situations. One such situation is a dead-ended narrow space, which can also exist in nature. If an organism is unable to retreat from the narrow space, this becomes a problem of survival. It is known that, to retreat from the narrow space, *Paramecia* fold their bodies very tightly and somehow turn even in narrow spaces (Smith, 1908; Day and Bentley, 1911). This is an emergence of new behavior. When the confined space was, however, too narrow in which to turn, *Paramecia* could perform LBS as shown in this report. This is another emergence of new behavior. This switching of behavioral options implies that *Paramecium* prefers forward swimming but a qualitatively different strategy emerges in the case of impossibility to turn. Such a long narrow space as tested in this report could be very rare in nature but the potential ability to evacuate from the long capillary is stored and appears when necessary. With respect to narrow space of capillary, emergence of new behavior occurs two times: turning by tightly folding and LBS. Although ciliate *Paramecia* are a single-celled organism, they can choose a better way from a variety of options to avoid difficult situations. A mechanism to switch these behaviors is an interesting theme from the point of view evolving behaviors and perhaps primitive intelligences.

It has been debated for many years whether ciliates have the capacity of associative learning as higher organisms do. When *Paramecium* or *Stentor* was confined in a vertically fixed capillary that was closed at the top and opened at the bottom in a water vessel, time to escape from the bottom of the capillary decreased after escape behaviors were performed repeatedly. Some researchers have claimed that this escape behavior was associative learning because two phenomena were associated: (1) “avoiding behavior” that was induced by the repetitive collisions to the top end, and (2) “reversal of swimming direction” that resulted in the success of escape (Bennett and Francis, 1972; Applewhite and Gardner, 1973; Huber et al., 1974). Contrary to this claim, Hinkle and Wood (1994) put forth a counter-argument that the escape behavior from the vertical capillary was not associative learning. The reason for their argument was because *Stentor* tended to gather at the bottom of capillary even if the exit of capillary was reversed from the bottom to the top of capillary. *Stentor* may just prefer to cluster at the lower ends of capillaries.

As the capillary was fixed horizontally in our experiment, the preference of vertical direction was not addressed for the escape movements of swimming organisms. However, our experimental setup is amenable to examination of associative learning, where, for instance, the latency of onset and offset of Paramecium escape behaviors may be modified by iterative learning experiences. According to our mathematical model proposed in this paper, such associative learning may be possible. Nevertheless, further studies are required to determine the temporal and mechanistic characteristics of any type of associative memory expression. In terms of conventional ethology, associative learning and memory might be expressed as operants and the formation of new behaviors. Although past studies have attempted to demonstrate operant conditioning in protozoa (Corning et al., 1973; Eisenstein, 1975), few have been successful when considering criteria established from animal research. But we anticipate the ethological significance of LBS will encourage constructive studies on the mechanisms of behavioral evolution, such as adaption or learning processes, and on the cytological physiochemical processes that may underlie them.

## Funding

This study was supported by the Strategic Japanese-Swedish Research Cooperative Program, JSPS KAKENHI Grant Number 25730178, and a Grant-in-Aid for Scientific Research on Innovative Areas from MEXT (25111726).

### Conflict of interest statement

The authors declare that the research was conducted in the absence of any commercial or financial relationships that could be construed as a potential conflict of interest.

## References

[B1] Applewhite P. B. Gardner F. T. (1973). Tube-escape behavior of paramecia. Behav. Biol. 9, 245–250 10.1016/S0091-6773(73)80159-2 4721217

[B2] Bennett D. A. Francis D. (1972). Learning in stentor. J. Eukaryotic Microbiol. 19, 484–487

[B3] Bray D. (2009). Wetware: A Computer in Every Living Cell. New Haven: Yale University Press

[B4] Corning W. C. Dyal J. A. Willows A. (1973). Invertebrate Learning: I. Protozoans Through Annelids. New York, NY: Plenum Press 10.1007/978-1-4684-3006-6_4

[B5] Day L. M. Bentley M. (1911). A note on learning in paramecium. J. Anim. Behav. 1, 67–73 10.1037/h0071290

[B6] Dunlap K. (1977). Localization of calcium channels in paramecium caudatum. J. Physiol. 271, 119–133 915829 10.1113/jphysiol.1977.sp011993 PMC1353610

[B7] Eckert R. (1972). Bioelectric control of ciliary activity. Science 176, 473–481 10.1126/science.176.4034.473 5032346

[B8] Eisenstein E. M. (1975). Aneural Organisms in Neurobiology. New York, NY: Springer 10.1007/978-1-4613-4473-5

[B9] Hamilton T. C. (1975). Behavioral plasticity in protozoans, in Aneural Organisms in Neurobiology, ed Eisenstein E. M. (New York, NY: Springer), 111–130 10.1007/978-1-4613-4473-5_8

[B10] Hennessey T. Kung C. (1985). Slow inactivation of the calcium current of paramecium is dependent on voltage and not internal calcium. J. Physiol. 365, 165–179 2411920 10.1113/jphysiol.1985.sp015765 PMC1192995

[B11] Hinkle D. J. Wood D. C. (1994). Is tube-escape learning by protozoa associative learning? Behav. Neurosci. 108, 94–99 10.1037/0735-7044.108.1.94 8192854

[B12] Hinrichsen R. D. Saimi Y. Kung C. (1984). Mutants with altered ca^2+^-channel properties in paramecium tetraurelia: isolation, characterization and genetic analysis. Genetics 108, 545–558 6094305 10.1093/genetics/108.3.545 PMC1202424

[B13] Hodgkin A. L. Huxley A. F. (1952). A quantitative description of membrane current and its application to conduction and excitation in nerve. J. Physiol. 117, 500–544 12991237 10.1113/jphysiol.1952.sp004764 PMC1392413

[B14] Huber J. C. Rucker W. B. McDiarmid C. G. (1974). Retention of escape training and activity changes in single paramecia. J. Comp. Physiol. Psychol. 86, 258–266 10.1037/h0035957

[B15] Machemer H. Machemer-Röhnisch S. (1984). Mechanical and electric correlates of mechanoreceptor activation of the ciliated tail inparamecium. J. Comp. Physiol. A 154, 273–278 10.1007/BF00604993

[B16] Machemer H. Ogura A. (1979). Ionic conductances of membranes in ciliated and deciliated paramecium. J. Physiol. 296, 49–60 529122 10.1113/jphysiol.1979.sp012990 PMC1279063

[B17] Naitoh Y. (1974). Bioelectric basis of behavior in protozoa. Am. Zool. 14, 883–893

[B18] Naitoh Y. (1990). Behavior of Unicellular Animals (in Japanese). Tokyo: University of Tokyo Press

[B19] Naitoh Y. Kaneko H. (1972). Reactivated triton-extracted models of paramecium: modification of ciliary movement by calcium ions. Science 176, 523–524 10.1126/science.176.4034.523 5032354

[B20] Naitoh Y. Sugino K. (1984). Ciliary movement and its control in paramecium1. J. Eukaryotic Microbiol. 31, 31–40

[B21] Ogura A. Machemer H. (1980). Distribution of mechanoreceptor channels in theparamecium surface membrane. J. Comp. Physiol. 135, 233–242 10.1007/BF00657251

[B22] Satow Y. MurphyN D. A. Kung C. (1983). The ionic basis of the depolarizing mechanoreceptor potential of paramecium tetraurelia. J. Exp. Biol. 103, 253–264

[B23] Smith S. (1908). The limits of educability in paramœcium. J. Comp. Neurol. Psychol. 18, 499–510 10.1002/cne.920180506

[B24] St John R. D. Corning P. A. (1973). Maternal aggression in mice. Behav. Biol. 9, 635–639 10.1016/S0091-6773(73)80058-6 4796850

[B25] Tominaga T. Naitoh Y. (1994). Comparison between thermoreceptor and mechanoreceptor currents in paramecium caudatum. J. Exp. Biol. 189, 117–131 9317455 10.1242/jeb.189.1.117

[B26] Wood D. C. (1975). Protozoa as models of stimulus transduction, in Aneural Organisms in Neurobiology, ed Eisenstein E. M. (New York, NY: Springer), 5–23 10.1007/978-1-4613-4473-5_2

